# Current Hospital Topics

**Published:** 1907-09-28

**Authors:** 


### HOSPITAL ADMINISTRATION.

### CONSTRUCTION AND ECONOMICS.

### CURRENT HOSPITAL TOPICS.

**The late Mr. R. G. Lund.**

It is with sincere regret and sorrow that we have to record the death of Mr. Reginald G. Lund, who for more than five years has fulfilled the duties of organising secretary to the League of Mercy. Previous to his appointment he held a similar post in connection with Charing Cross Hospital, and was the means of bringing considerable funds to the coffers of that institution. Mr. Lund was untiring in his labours on behalf of the hospitals through the League of Mercy; he had an exceedingly pleasant manner, and was deservedly popular with a multitude of people in all ranks of society with whom he had to deal in connection with an office where personal charm, and the tactful knowledge of individuals, were gifts of momentous importance. Mr. Lund believed in the doctrine of personal service in the cause of the sick in the days of health, and afforded practical proof of this fact by the zeal with which he fulfilled the duties of his office. It is grievous to remember that Mr. Lund, who was in the prime of life, should have been suddenly attacked early in August, that he should have spent the greater part of that month in Charing Cross Hospital, that he should have undergone two operations, and that, on his leaving in a convalescent state early in September, he should have had to return to a hospital bed within a very short time, and should have succumbed to an anæsthetic which was given to enable the surgeons to make a preliminary examination of the causes of the new trouble which had returned him on their hands. The League of Mercy exists to enable thoughtful people to render praise for the blessings of health in a practical manner. The sudden and tragic death of Mr. Lund must bring home to every one of the members the uncertainty of life, and will, we have little doubt, make them even more zealous in the future than they have been in the past in the cause of the suffering and the sick. Our sympathies go out to Mrs. Lund and the children, and we venture to hope that steps may be taken to recognise the good work done by Mr. Lund during his life, and in some measure, if it be possible, to lighten the heavy burden of care which must fall on the widow and the fatherless.

**The Sheffield Union Hospital Scandal.**

In our issues of April 27 and May 4 last, on the occasion of the resignation of three medical officers attached to this institution, to which the *Sheffield Independent* rightly called attention, we ventured to say that it was clear that "there must be something fundamentally wrong with the attitude of the hospital authorities towards the resident medical staff." At that time eight resignations of medical officers had taken place, which we pointed out at the time proved that the relations between the resident medical staff and the local Guardians were not such as they should be. The essentials of the fit administration of a great hospital were not apparently understood by the Guardians of the Sheffield Union, and for this reason we demanded inquiry and reform. We received a letter from a member of the Hospital Committee at the time, in which he deprecated criticism, and urged that everything was being done that could be done to make the position of resident medical officer satisfactory and comfortable.

We gather that the crux of the whole matter has from the first lain in the fact that the Hospital Committee have declined to define the terms of appointment and the duties of the medical staff. There seems to have been a spirit of something like jealousy exhibited by the Hospital Committee in respect to the authority vested in the medical officer by the Local Government Board by their general Order dated March 15, 1906. That Order makes it perfectly clear that the medical officer is to have the control of the whole of the staff, as well as of the patients, together with the arrangements and administration of the wards and all that appertains thereto. The Hospital Committee, however, appear to think that it is not necessary, for instance, to consult the medical officer before some other official takes it on himself to remove a patient from one bed to another, or even from one ward to another, and that, in matters other than treatment, officials other than the medical officer should be left to follow their own will, independent of any medical control. If, as we understand, any such state of affairs as this has been allowed to grow up, despite the Order of the Local Government Board to which we have just referred, then it is certain that steps should speedily be taken to dissolve the existing Hospital Committee and to substitute in its place men with administrative ability and business judgment, who will take care in future not only that the orders of the Local Government Board in respect to their officers are precisely followed, but that the internal administration of the Sheffield Union Hospital shall be placed under the sole authority of the medical superintendent as the supreme head, and that he shall be held responsible for the efficiency of each department and for the comfort, care and cure of all the patients within its walls.

When resignations first became the fashion at this institution it was contended, on behalf of the Hospital Committee, that, although the matter was brought to the notice of the Executive Committee of the Sheffield division of the Yorkshire branch of the British Medical Association, the latter committee determined "that the matter did not travel beyond domesticity, and was one that did not call for their attention." We should be glad to hear whether this contention on behalf of the Hospital Committee is correct or not. If it is, it seems to us a little unfortunate that more care was not taken to go thoroughly into the matter, for, had this course been followed, the existing grave scandal might have been spared, with the consequent risk to the well-being, and even possibly to the lives, of some upwards of four hundred patients, who are apparently at the moment in danger of being left without any medical care or supervision whatever. Certainly the present state of affairs is not creditable to the administrative abilities of the Guardians of Sheffield, who will find themselves in a position of grave responsibility, if they have four or five hundred sick poor on their hands without being able to obtain medical assistance for them.

The Local Government Board, in consequence of the resignation of the whole of the medical staff, both resident and visiting, have agreed to send an inspector to investigate the position of affairs which has created such unbearable friction between the medical staff and the Hospital Committee of the Board of Guardians, in regard to the position of the resident medical officers. We hope that the inspector will set to work with a will, and that in the result a Hospital Committee may be selected of adequate capacity to undertake its responsible duties as the executive head of a great Poor-law hospital like that of the Sheffield Union.

